# Introductory Lecture

**Published:** 1853-04

**Authors:** 


*Introductory Lecture,* by Professor J. G. HUGHES, *before the Class of Medical Students at the opening of the session of* 1852-'3, *of the College of Physicians, and Surgeons of the Iowa University.*

THIS address exhibits a good deal of learning and research, in pointing out the past medical follies, and in endeavoring to show the necessity of settled principles, and of having fixed elements of truth, which are termed the "basis element," as the only trust-worthy foundation in solid advancement in any science or art, medical as well as every other.

